# Balanced Centrality of Networks

**DOI:** 10.1155/2014/871038

**Published:** 2014-11-03

**Authors:** Mark Debono, Josef Lauri, Irene Sciriha

**Affiliations:** Department of Mathematics, Faculty of Science, University of Malta Msida, MSD 2080, Malta

## Abstract

There is an age-old question in all branches of network analysis. What makes an actor in a network important, courted, or sought? Both Crossley and Bonacich contend that rather than its intrinsic wealth or value, an actor's status lies in the structures of its interactions with other actors. Since pairwise relation data in a network can be stored in a two-dimensional array or matrix, graph theory and linear algebra lend themselves as great tools to gauge the centrality (interpreted as importance, power, or popularity, depending on the purpose of the network) of each actor. We express known and new centralities in terms of only two matrices associated with the network. We show that derivations of these expressions can be handled exclusively through the main eigenvectors (not orthogonal to the all-one vector) associated with the adjacency matrix. We also propose a centrality vector (SWIPD) which is a linear combination of the square, walk, power, and degree centrality vectors with weightings of the various centralities depending on the purpose of the network. By comparing actors' scores for various weightings, a clear understanding of which actors are most central is obtained. Moreover, for threshold networks, the (SWIPD) measure turns out to be independent of the weightings.

## 1. Introduction

An actor is represented by a vertex *i* in a relational network. Following the terminology used in social analysis, the power and importance of a specific actor (*ego*) does not result from its inherent properties but rather from its position with respect to others (*alter*) in the network. The extent of its influence depends on the links to first neighbours and the neighbours of these neighbours. What is relevant is the importance of the actors in the subnetwork focused around* ego*, mostly that including all actors to whom ego has a connection up to a prescribed path length, and all the connections among all of these actors.

To rank actors according to their importance, one can gauge the amount of their influence in the whole network. There are various types of centralities of an actor *i* in a relational network. A* centrality* measure is meant to give the relative importance of *i* in the network. It is usually taken to be intuitively a measure of the access of *i* to sources and the power *i* can wield to disseminate ideas and create awareness. We will also quantify a new aspect of power depending on the extent* alter* is constrained to depend on* ego* for the purpose of their membership in the network. For any specified centrality measure, the vector with the *i*th entry equal to the centrality of the vertex *i* is said to be the* centrality vector* of the network.

A network is represented by a graph (*G*, *𝒱*, *ℰ*) with a set *𝒱* of vertices often labelled 1,2,…, *n* and an edge-set *ℰ* of pairs of distinct vertices describing an adjacency relation. Network systems are modelled as graphs whose vertices represent the dynamical units or actors and whose links stand for the interactions (collaborations and business links for instance) among them. Powerful graph theoretical techniques can then be applied to yield results that give meaningful information about a network.

The* degree ρ*_*i*_ of a vertex *i* is the number of edges incident to *i*. A vertex of degree one is termed an* end-vertex*. In a *ρ*-regular graph, *ρ*_*i*_ has the same value *ρ* for each vertex *i*. A* walk* of length *k* starting from a vertex *i* is an alternating sequence of (not necessarily distinct) vertices and edges *v*_1_, *e*_1_, *v*_2_, *e*_2_,…, *e*_*k*_, *v*_*k*+1_ where *v*_1_ = *i* and an edge *e*_*ℓ*_ has *v*_*ℓ*_ and *v*_*ℓ*+1_ as end vertices. A* clique* is a subset of the vertices that induces a complete subgraph. An* independent set* is a subset of the vertices that induces an empty subgraph (with no edges). The* path P*_*n*_ of length *n* − 1 is an alternating sequence *v*_1_, *e*_1_, *v*_2_, *e*_2_, *v*_3_,…, *v*_*n*_ of* distinct* vertices *v*_1_, *v*_2_,…, *v*_*n*_ and distinct edges *e*_1_, *e*_2_,…, *e*_*n*−1_ so that *e*_*i*_ is an edge connecting *v*_*i*_ to *v*_*i*+1_. The* distance* between two vertices *v*_*i*_ and *v*_*j*_ is the number of edges in the shortest path joining *v*_*i*_ to *v*_*j*_. The maximum of the distances between all the vertex pairs in *G* is called the* diameter* of *G*.

The* cycle C*_*n*_ is a connected graph on *n* vertices each with degree two. The* complete graph K*_*n*_ has *n* vertices and there is an edge between every pair of vertices. It follows that the vertices of a complete graph form a clique. In a* bipartite graph*, the vertex set is partitioned into independent sets *𝒱*_1_ and *𝒱*_2_.

The purpose of a network determines which centrality measures are meaningful. Information networks aim to reach as many actors as possible, in contrast with epidemiology networks whose objective is to contain the spread of a virus. In exchange networks, bargaining power is the goal and* ego*'s power is alter's dependence. To strike a balance among the intended behaviour and the latent forces emanating from the network structure, we consider various centrality measures, namely,

- the degree centrality,
- the square centrality,
- the eigenvector centrality,
- the walk probability vector,
- the walk centrality,
- the irregular scaled-walk centrality,
- the power centrality.

Measures (i) and (iii) are standard centralities, whereas the rest are inspired by behavioural network expectations. The local statistics of a vertex are captured, for instance, by its degree or by the entry, corresponding to a particular vertex, of a specific eigenvector for some matrix encoding the graph adjacencies. We propose another general-purpose centrality termed SWIPD which is a linear combination of the square, walk, power, and degree centrality vectors where weightings can be varied depending on the overarching aim of the network.

We use **A**(*G*) (or just **A** when the context is clear) to denote the 0-1-*adjacency matrix* of a graph *G*, where the entry *a*_*ik*_ of the symmetric matrix **A** is 1 if {*i*, *k*} ∈ *ℰ* and 0 otherwise. We note that the graph *G* is determined, up to isomorphism, by **A**. For a real symmetric matrix **M**, the real number *λ* is an eigenvalue of a matrix **M** if there exists a nonzero vector **x** (termed a *λ*-eigenvector) satisfying **M** **x** = *λ* **x**. The *λ*-*eigenspace* is the subspace containing all the *λ*-eigenvectors. The* nullity* of **M** is the multiplicity of the eigenvalue zero of **M**. It can also be seen as the deficiency in the* rank* of **M**.

The vector **j** ∈ *ℝ* ^*n*^ with each entry equal to one indicates that all vertices have identical weights. The eigenvalues of the adjacency matrix of a graph *G* having some eigenvector* not* orthogonal to **j** are said to be the* main eigenvalues* of *G*. A regular graph *G* has **j** as an eigenvector and therefore it has only one main eigenvalue, namely, the maximum eigenvalue. The vector **j** is used as an initial vertex status in a bias-free network.

By the* Perron-Frobenius theorem* on nonnegative matrices, the adjacency matrix **A** of a connected network has an eigenvector each of whose entries is positive. This eigenvector is referred to as the* Perron vector* and is associated with the maximum eigenvalue of **A** (or of *G*). Note that the maximum eigenvalue *λ*_max⁡_ of **A** is not exceeded by the absolute value of any other eigenvalue and is always a main eigenvalue.

The* degree diagonal matrix* **D** has *ρ*_*i*_ as the diagonal entry at position *i* for 1 ≤ *i* ≤ *n* and zero in all off-diagonal positions. A very useful representation of a graph *G* is the* Laplacian matrix* (**L** **a** **p**) defined as **D** − **A**. For a connected network, the Laplacian *Lap* has a simple eigenvalue zero, with an associated eigenvector equal to **j**.

An attractive feature in the mathematical treatment of the formulae for the centralities derived here is that

(i) the main eigenvalues suffice in all the derivations and proofs;
(ii) each centrality measure is expressed in terms of the matrices **D** and **A** only, which renders the computations tractable.

The rest of the paper will be organised as follows. In Section 2, we review the established degree and eigenvector centralities of a network *G* and refine them by expressing them in terms of invariant vectors associated with **A**. We also introduce the square centrality that focuses on the subnetwork of actors that are at a distance at most two from *ego*. Networks often model the spread of fluids, the diffusion of information, or virus propagation.* Diffusion* is governed by a differential equation that can be expressed in terms of the Laplacian. In algorithms that determine network kinetics, the Laplacian is a recurring theme. We show, in Section 3, how the Laplacian features in the diffusion of information, which is found to rank vertices as the degree centrality does. We then propose, in Section 4, the walk centrality vector, based on the main eigenspaces and, later, the power centrality that reduces the contribution to* ego* by the well connected first neighbours.

**Figure 1:**
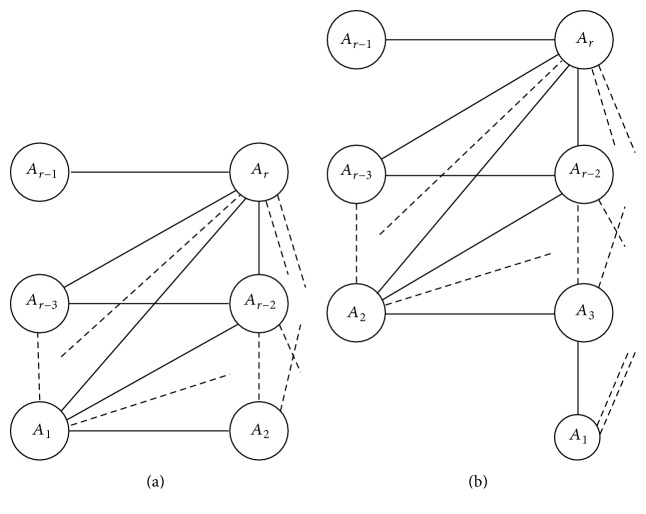
The threshold graphs *C*(*a*_1_, *a*_2_,…, *a*_*r*_), |*A*_*i*_| = *a*_*i*_, *a*_*i*_ ≥ 2, for *r* even and *r* odd, respectively.

A* split graph* is a graph in which the vertex set can be partitioned into a clique and an independent set. A connected* threshold graph C*(*a*_1_, *a*_2_,…, *a*_*r*_) is a split graph in which the independent subset (if not empty) is partitioned into one or more parts *A*_*r*−1_, *A*_*r*−3_,…, *A*_*t*_ and the clique is partitioned into one or more parts *A*_*r*_, *A*_*r*−2_,…, *A*_*s*_ where *t* = 1 and *s* = 2 if *r* is even, whereas *t* = 2 and *s* = 1 if *r* is odd, as shown in Figure 1. Note that |*A*_*i*_| = *a*_*i*_ ≥ 1 and, for a unique representation *C*(*a*_1_, *a*_2_,…, *a*_*r*_), the size |*A*_1_| = *a*_1_ ≥ 2. Each vertex of a particular independent subset *A*_*i*_ has the same neighbourhood *A*_*i*+1_ ∪ *A*_*i*+3_ ∪ ⋯∪*A*_*r*_. The *r* distinct vertex degrees are *ρ*_1_, *ρ*_2_,…, *ρ*_*r*_. Moreover the closed neighbourhood of a vertex contains the neighbourhood of any vertex of lower degree, which is the reason why threshold graphs are also referred to as* nested split graphs*. The interactions within many real world networks approach those of threshold graphs [1, 2]. In the sequel, we will point out instances when threshold graphs show limiting behaviour for the centrality measures we consider. In Section 5, we establish that, for threshold graphs, even the expected discriminating centralities such as the eigenvector and the power centrality coincide with the degree centrality.

In Section 4.2 we discuss the graph parameter (SWIPD) that combines the centralities that tend to provide unique information on a network. Only for irregular nonbipartite graphs does the SWIPD centrality give a meaningful ranking of the vertices.

## 2. Local Centralities

The most central vertices in a network are expected to head the list in a valid ranking of the vertices. At a first level of* ego's* exposure, one may look at the immediate neighbours, captured by the* degree centrality measure*. The status of these first neighbours is also thought to be of significance to* ego's* ranking as they may bring their influence to bear on the rest of the network according to* ego's* needs. We therefore consider three aspects of the influence of vertices at a distance up to two from* ego*. Firstly, the number of the immediate neighbours in the degree centrality, secondly the total number of first and second neighbours of* ego* will be considered in the* square centrality,* and thirdly the ripple effect of the first neighbours' own centrality covered by the* eigenvector centrality*.

### 2.1. Degree and Square Centrality Measures

An accepted premise, in propagation networks, is that the popularity of an actor increases with the number of links to others in the network. The* degree centrality* of a vertex *i* is its degree *ρ*_*i*_, that is, the total number of its immediate neighbours. The vector **d** with entry *i* equal to the degree centrality of *i* is said to be the* degree centrality vector* of the network. Recall that the vector **j** = (1,1,…, 1)^*t*^ gives equal importance to all the vertices.

**Proposition 1 .**

Let *G* be a network with adjacency matrix **A**. The degree centrality vector is **A** **j**.

**Proof**

Let *ρ*_*i*_ be the degree of vertex *i*. Then $Aj={\left(\begin{array}{c} \rho_{1},\rho_{2},\ldots ,\rho_{n} \end{array}\right)}^{t}=d$, as required.

The degree centrality is a main contributor to vertex centrality. In Section 3 we will see that there are other centrality measures which are also proportional to **A** **j**, for any graph. One of them, in particular, ranks vertices according to the likelihood that a random walk ends at a particular vertex.

The degree sequence is only the first level of understanding of* ego*'s status. The next step is to consider implicit interactions of* alter* with* ego*. One may ask whether the number of actors at distance two or more have a significant effect on* ego*. The rationale for this approach is that the formation of new links in a network, where dissemination of information is a priority, tends to be influenced by the choices of the first neighbours. We expect links with “friends of friends” to be more likely. The sum of the first and second neighbours from each vertex is given by the vector **A** ^2^ **j**.

Large distances between two actors in a network do not necessarily exclude mutual influence. Exposure to information received by different actors even if not directly from* ego* may have a significant impact on them. The same goes for “similar” information sent by different actors at various distances from* ego* and received by* ego* through interaction, which often leads to progressing stages of empathy, a sense of familiarity with the information and acceptance, possibly leading to a proper understanding of (or yearning for) it. Seen in another light, consensus for the acceptance of a new product by many actors in a network is necessarily reached by means of such interactions. The centrality measures we will now study take into consideration distance of* alter* from* ego* throughout the network.

### 2.2. Eigenvector Centrality

We now look at the second aspect of the influence of neighbours, that is, the ripple effect of first neighbours' own centrality on* ego*'s ranking. Effective measures aimed at increasing* ego's* status are important in marketing. Wasserman and Faust [3] discuss what they call prestige measures of centrality, that is, measures in which the centralities (statuses) of positions are recursively related to the centralities of the positions to which they are connected. Instead of just looking at the vertex degree of a specific actor *ego* and of its immediate neighbours as an indication of* ego*'s importance in a network, the influence of its neighbours is also considered. The interpretation is that having a neighbour who has power over others adds to* ego*'s importance. Moreover, links are often made with actors recommended by a neighbour. A measure *𝒞*_*i*_ of centrality based on the centralities of neighbours is achieved by assigning a weight to each vertex *i* equal to its interim centrality in an iteration converging to *𝒞*_*i*_. Whereas degree centrality gives every contact the same weight, the eigenvector centrality weights link with others according to their centralities, thus taking into account the entire pattern in the network. Since repeated application of the adjacency matrix **A** on a vector increases the value exponentially when the maximum eigenvalue *λ*_max⁡_ of **A** exceeds 1, control is achieved by scaling **A** to (1/*λ*_max⁡_)**A**.

**Definition 2 .**

Let **y**_*r*_ = (1/*λ*_max⁡_)**A** **y**_*r*−1_, *y*_0_ = **j**, and let lim⁡_*r*→*∞*_ **y**_*r*_ = **y**. Then the* eigenvector centrality* $\hat{y}$ is the unit vector along **y**.

Starting with the vector **j** ensures a bias-free process. The eigenvalues of a diagonalizable matrix **A** whose eigenvectors span **j** are said to be* main*. If **A** is real and symmetric and has *s* distinct eigenvalues *μ*_1_, *μ*_2_,…, *μ*_*s*_, then diagonalization leads to the spectral decomposition **A** = *μ*_1_ **P**_1_ + *μ*_2_ **P**_2_ + ⋯+*μ*_*s*_ **P**_*s*_, where **P**_*i*_ is the projection onto the eigenspace of *μ*_*i*_, for 1 ≤ *i* ≤ *s*.

**Lemma 3 (see [4]).**

If **P**_1_, **P**_2_,…, **P**_*s*_ are the projections onto the **A**-eigenspaces of the *s* distinct eigenvalues *μ*_1_, *μ*_2_,…, *μ*_*s*_, respectively, of **A**, then ∑_*i*=1_^*s*^ **P**_*i*_ is the identity operator **I**.

Let *μ*_1_, *μ*_2_,…, *μ*_*p*_ be the main eigenvalues of *G*, written in monotonic decreasing order, where 1 ≤ *p* ≤ *s*. From Lemma 3 and the definition of main eigenvalues, we can write **j** = ∑_*i*=1_^*p*^ **P**_*i*_ **j**. Thus **j** can be expressed as the sum of *p* orthonormal eigenvectors {**P**_1_ **j**, **P**_2_ **j**,…, **P**_*p*_ **j**} of *G* associated with the main eigenvalues *μ*_1_, *μ*_2_,…, *μ*_*p*_, respectively.

**Lemma 4 .**

If **z** ^(1)^, **z** ^(2)^,…, **z** ^(*p*)^ are the unit vectors along **P**_1_ **j**, **P**_2_ **j**,…, **P**_*p*_ **j**, respectively, then **j** = ∑_*i*=1_^*p*^ *β*_*i*_ **z** ^(*i*)^ where *β*_*i*_ = ‖**P**_*i*_ **j**‖ ≠ 0 for each *i*, 1 ≤ *i* ≤ *p*.

The unique vectors **z** ^(1)^, **z** ^(2)^,…, **z** ^(*p*)^ of Lemma 4 are referred to as the* main eigenvectors* of *G* [5, 6].

**Lemma 5 (see [7]).**

Let *G* be a graph with *p* main eigenvalues *μ*_1_, *μ*_2_,…, *μ*_*p*_. The total number *N*_*k*_ of walks of length *k* is given by *N*_*k*_ = ∑_*i*=1_^*p*^ *c*_*i*_′*μ*_*i*_^*k*^, where for 1 ≤ *i* ≤ *p*, the scalars *c*_*i*_′ = ‖**P**_*i*_ **j**‖^2^ are independent of *k*.

The iteration defining the eigenvector centrality in Definition 2 is known as the* power method*. It converges provided the network *G* is not bipartite (that is provided *G* has an odd cycle) (see [8], e.g.). We give a proof using only the main eigenvalues.

**Theorem 6 .**

For a nonbipartite graph, the eigenvector centrality $\hat{y}$ is the unit Perron vector of **A**.

**Proof**

Consider the iteration **y**_*r*_ = (1/*λ*_max⁡_)**A** **y**_*r*−1_ where **y**_0_ = **j**. From Lemma 4, **j** = *β*_1_ **z** ^(1)^ + *β*_2_ **z** ^(2)^ + ⋯+*β*_*p*_ **z** ^(*p*)^ where **z** ^(1)^, **z** ^(2)^,…, **z** ^(*p*)^ are orthonormal eigenvectors belonging to the main eigenvalues *μ*_1_, *μ*_2_,…, *μ*_*p*_. For a nonbipartite connected graph, *μ*_1_ is the maximum eigenvalue *λ*_max⁡_ of **A** and is larger than the absolute value of all the other eigenvalues. From the eigenvector equations **A** **z** ^(*i*)^ = *λ*_*i*_ **z** ^(*i*)^ and **y**_*r*_ = (1/(*λ*_max⁡_)^*r*^)**A** ^*r*^ **y**_0_, we obtain **y**_*r*_ = ∑_*i*=1_^*p*^ *β*_*i*_(*μ*_*i*_/*λ*_max⁡_)^*r*^ **z**_*i*_. As *r* → *∞*, (*μ*_*i*_/*λ*_max⁡_)^*r*^ → 0 for *μ*_*i*_ < *λ*_max⁡_ and **y**_*r*_ tends to a limit proportional to the Perron vector **z** ^(1)^.

We note that for bipartite graphs, however, the minimum eigenvalue *λ*_*n*_ of **A** is −*λ*_max⁡_ and if it happens to be main, then the iteration oscillates as *r* → *∞*.

For many graphs, the eigenvector centrality captures properties of second neighbours and gives a vertex ranking often different from the degree centrality ranking [9]. This is not the case for threshold graphs.

**Proposition 7 (see [10]).**

For threshold graphs, the ranking of vertices according to the eigenvector centrality is equal to that according to the degree centrality.

## 3. The Discrete Laplacian and Walks

Understanding the specific details of interactions with* ego* is an essential part of figuring out the factors that may increase* ego*'s status. When direct methods prove difficult, a complementary approach is provided by discovering network substructures and invariants that also play a key role.

A graph invariant that goes beyond the immediate neighbourhood of* ego* is distance between* ego* and a vertex in* alter*. The investigation of random movement along a network often involves the Laplacian matrix. In the physical theory of diffusion, the Laplacian arises naturally in the mathematical analysis leading to the equilibrium state.

### 3.1. Propagation

*Diffusion* can be seen as the random motion of fluid particles from regions of higher concentration to regions of lower concentration. The same terminology is borrowed for the spread of a commodity *χ* such as information or disease. The rate of diffusion *dχ*_*i*_/*dt* from a vertex *i* in a network depends on the difference in the amounts of commodity between *i* and its neighbours. Thus *dχ*_*i*_/*dt* = *κ*∑_*j*_ *a*_*ij*_(*χ*_*j*_ − *χ*_*i*_), for a diffusion constant *κ*.

**Definition 8 .**

If the amount of diffusing commodity at a vertex *i* in a network is *χ*_*i*_, then the column vector **X** = (*χ*_1_, *χ*_2_,…, *χ*_*n*_)^*t*^ is said to be the* diffusion vector*.

We give a proof of the following well known result that links the Laplacian with the discrete Laplacian.

**Proposition 9 .**

The differential equation regulating diffusion in a network is *d* **X**/*dt* = −*κ* **L** **a** **p** **X**.

**Proof**

Let *δ*_*ij*_ be the Kronecker delta which is equal to one if *i* = *j* and zero otherwise. Since *dχ*_*i*_/*dt* = *κ*∑_*j*_ *a*_*ij*_(*χ*_*j*_ − *χ*_*i*_) = *κ*(∑_*j*_(*a*_*ij*_ *χ*_*j*_) − *χ*_*i*_ *ρ*_*i*_) = *κ*∑_*j*_(*a*_*ij*_ − *δ*_*ij*_ *ρ*_*j*_)*χ*_*j*_, then

(1) $$\begin{array}{c} \frac{dX}{dt}=\kappa \left(A-D\right)X=-\kappa Lap\;\;X. \end{array}$$

The well known diffusion equation is *d* **X**/*dt* = *κ*∇^2^ **X** where ∇^2^ is the Laplacian operator. For this reason, the matrix **L** **a** **p** = **D** − **A** is referred to as the* discrete Laplacian* of the graph. The solution of (1) is obtained by expressing **X** as a linear combination of the *n* orthonormal eigenvectors {**v**_*i*_} corresponding to the eigenvalues of the real and symmetric Laplacian **D** − **A**. Thus **X**(*t*) = ∑_*i*=1_^*n*^ *α*_*i*_(*t*)**v**_*i*_. Substituting in (1), (*d*/*dt*)∑_*i*=1_^*n*^ *α*_*i*_(*t*)**v**_*i*_ = −*κ*∑_*i*=1_^*n*^ *α*_*i*_(*t*)**L** **a** **p** **v**_*i*_ = −*κ*∑_*i*=1_^*n*^ *α*_*i*_(*t*)*μ*_*i*_ **v**_*i*_. Solving (*d*/*dt*)*α*_*i*_(*t*) = −*κα*_*i*_(*t*)*μ*_*i*_ gives the exponential decay *α*_*i*_(*t*) = *α*_*i*_(0)*e* ^−*κμ*_*i*_*t*^ for initial commodity amounts {*α*_*i*_(0)}.

### 3.2. The Walk Probability Vector

In a random walk, or Markov chain, along the edges of a connected graph *G* with adjacency matrix **A** = (*a*_*ik*_), starting from a particular vertex, the probability *p*_*i*_(*θ*) that a walker is at vertex *i*, after traversing *θ* edges (or at time *θ*), the sum over all the neighbours of *i* of *p*_*j*_(*θ* − 1)*p*(*i*_*θ*_∣*j*_*θ*−1_) where *p*(*i*_*θ*_∣*j*_*θ*−1_) is the probability that the walker moves along edge {*j*, *i*}, given that it is at *j* after time *θ* − 1. The unbiased probability *p*(*i*_*θ*_∣*j*_*θ*−1_) is 1/*ρ*_*j*_. The adjacency matrix entries are used to select only the neighbours *j* of *i*. Therefore

(2) $$\begin{array}{c} p_{i}\left(\theta \right)=\underset{k}{\sum }a_{ik}\frac{1}{\rho_{k}}p_{k}\left(\theta -1\right), \end{array}$$

which can be expressed in terms of the Laplacian, as will be shown in Corollary 12.

**Definition 10 .**

The column vector **p**(*θ*) = (*p*_1_(*θ*), *p*_2_(*θ*),…, *p*_*n*_(*θ*))^*⊤*^ is said to be the* walk probability vector at time θ*. In the limit, as *θ* → *∞*, the iteration converges and **p**(*θ*) approaches the* walk probability vector* **p**.

The diagonal matrix with 1/*ρ*_*i*_ as the diagonal entry at position *i* is **D** ^−1^. The entries of the *k*th column of **A** **D** ^−1^ are obtained by dividing the entries of the *k*th column of **A** by *ρ*_*k*_. Expression (2) can be simplified immediately as in the following result.

**Lemma 11 .**

(i) The* walk probability vector* **p**(*θ*) = **A** **D** ^−1^ **p**(*θ* − 1).

(ii) In the limit, as *θ* → *∞*, **p**(*θ*) → **p** and **p** = **A** **D** ^−1^ **p**.

The following results follow immediately.

**Corollary 12 .**

Consider the following:

(i) (**I** − **A** **D** ^−1^)**p** = 0;

(ii) (**I** − **A** **D** ^−1^) = **L** **a** **p** **D** ^−1^.

Let **d** be the vector with the vertex degree *ρ*_*i*_ as the entry at position *i*. We observe that

(i) in a* directed* graph, the matrix used in the iteration **y** = **A**(**D**′)^−1^ **y** + *β* **j** is that for* PageRank* where **D**′ is obtained from **D** modified by replacing each zero entries on its diagonal by one, so that it is invertible;
(ii) from Corollary 12, **D** ^−1^ **p** is in the nullspace of the Laplacian** Lap** and **D** ^−1^ **d** = **j**;
(iii) **D** ^−1^ **A** **j** = **j** and the matrix **D** ^−1^ **A** is referred to as the* transition matrix for a random walk* [11];
(iv) the Perron vector of **A** **D** ^−1^ is **d** for the maximum eigenvalue of **A**, which is 1.

**Theorem 13 .**

The* walk probability vector* **p**, for a connected network with *m* edges, is (1/2*m*)**A** **j**.

**Proof**

Since (**I** − **A** **D** ^−1^) = **L** **a** **p** **D** ^−1^, from Corollary 12(i), **L** **a** **p** **D** ^−1^ **p** = 0. For a connected network, the Laplacian is singular of nullity one, with the eigenvector **j** generating the zero-eigenspace. Hence **D** ^−1^ **p** is a multiple of **j**. Thus **p** = *α* **D** **j** = *α* **A** **j** for *α* ∈ *ℝ*. If **p** = (*p*_1_, *p*_2_,…, *p*_*n*_)^*⊤*^, since **D** is the diagonal matrix with *ρ*_*i*_ as the diagonal entry at position *i* and ∑_*i*=1_^*n*^ *p*_*i*_ = 1, then *α* **j** ^*⊤*^ **A** **j** = 1. Therefore *α* = 1/2*m*.

Theorem 13 gives a surprising result. It asserts that the* walk probability vector* that is designed to take into account the limiting behaviour of even the remote actors into consideration gives the same ranking of the vertices as the degree centrality.

## 4. Walk and Power Centralities

Although local properties are mostly influenced by immediate neighbourhoods, the relative importance of all actors in a network that can affect* ego* must be considered. The questions we wish to answer are as follows.

(i) What is the extent of influence on *ego* of remote actors?
(ii) Which centrality measures ensure that the impact on *ego*'s centrality, of the actors at a large distance from *ego*, is not ignored?

To answer these questions, we discuss a centrality that includes all actors in a network, based on the number of walks. However, the more remote actors are made to exert less influence on* ego* in this measure by applying a geometric progression scaling factor (see Definition 14).

### 4.1. Walk Centrality

In marketing, it is a common belief that a certain threshold density of exposure to a message is required to achieve effective communication. The larger the number of walks of various lengths *r* from a vertex *i* is, the more the possibilities *i* has to be influenced by actors that can reach it by traversing *r* edges (possibly repeated).

The *i*th entries of the vectors, **j**, **A** **j**, **A** ^2^ **j**,…, give the number of walks of length 0,1, 2,…, respectively, starting at vertex *i*. For an* attenuation* or* damping* factor *α* < 1, consider **y** = **j** + *α* **A** **j** + *α* ^2^ **A** ^2^ **j** + ⋯, where the number of walks of length *r* are scaled down by *α* ^*r*^. Similar measures have been proposed in [12, 13] by Bonacich and Katz, respectively.

**Definition 14 .**

Let *λ*_max⁡_ be the maximum eigenvalue of the adjacency matrix **A** of a graph *G*. For *α* < 1/*λ*_max⁡_, the unit vector along **y** = **j** + *α* **A** **j** + *α* ^2^ **A** ^2^ **j** + ⋯+*α* ^*r*^ **A** ^*r*^ **j** + ⋯ is said to be the* walk centrality vector*.

**Lemma 15 .**

If *α* < 1/*λ*_max⁡_, then (**I** − *α* **A**) is invertible.

**Proof**

Consider the determinant *δ* of the *n* × *n* matrix (**I** − *α* **A**). We can write *δ* as *α* ^*n*^det⁡((*α*)^−1^ **I** − **A**). By Perron-Frobenius theorem for a nonnegative matrix **A**, the absolute value of each eigenvalue of **A** does not exceed the maximum eigenvalue *λ*_max⁡_ of **A**. The characteristic polynomial det⁡(*λ* **I** − **A**) is of degree *n* and is positive for *λ* > *λ*_max⁡_. Thus for (*α*)^−1^ > *λ*_max⁡_, *δ* > 0 and therefore (**I** − *α* **A**) is invertible.

The walk centrality vector is defined for all *α* except at the eigenvalues of **A** ^−1^. The operator (**I** − *α* **A**)^−1^ is referred to as the* resolvent*, usually used in the study of the spectrum of operators on Hilbert spaces and applied to solve the inhomogeneous Fredholm integral equations via the Liouville-Neumann series. We now present new formulae for the walk centrality by considering only the main eigenvalues and eigenvectors of the adjacency matrix.

**Theorem 16 .**

Let *λ*_max⁡_ be the maximum eigenvalue of the *n* × *n* adjacency matrix **A** with corresponding Perron vector **z** ^(1)^. Then the walk centrality vector is the unit vector along **y** = (**I** − *α* **A**)^−1^ **j** which is

(3) $$\begin{array}{c} \frac{j}{\sqrt{n}}\;if\;\;\alpha ⟶0;\\ z^{\left(1\right)}\;if\;\;\alpha ⟶\frac{1}{\lambda_{\max}}. \end{array}$$

**Proof**

Let *μ*_1_(=*λ*_max⁡_), *μ*_2_,…, *μ*_*p*_ be the main eigenvalues of *G* with corresponding eigenvectors **z** ^(1)^, **z** ^(2)^,…, **z** ^(*n*)^ as in Lemma 4. Then,

(4) I−αA−1jW=∑k=0∞αkAkj=∑k=0∞αkAk‍∑i=1pβiz(i)W=∑k=0∞αk∑i=1pβiμikz(i)W=∑k=0∞αkβ1μ1kz1+β2μ2kz2+⋯+βpμpkzp.

Since *α* < 1/*λ*_max⁡_, for 1 ≤ *i* ≤ *p*, ∑_*k*=0_^*∞*^ *α* ^*k*^ *μ*_*i*_^*k*^ converges absolutely.

It follows that

(5) I−αA−1jW=β1∑k=0∞‍αμ1kz(1)+β2∑k=0∞‍αμ2kz(2)+⋯WW+βp∑k=0∞αμpkz(p)W=β1z(1)(1−αμ1)+β2z(2)(1−αμ2)+⋯+βpz(p)(1−μpα)W=11−αμ1WW×β1z1+1−αμ11−αμ2β2z2+⋯+1−αμ11−αμpβpzp.

As *α* → 0,  (**I** − *α* **A**)^−1^ **j** → **j**. Since lim⁡_*αμ*_1_→1_((1 − *αμ*_1_)/(1 − *αμ*_*i*_)) = 0 for 2 ≤ *i* ≤ *p*, then

(6) $$\begin{array}{c} \underset{\alpha \mu_{1}\to 1}{\lim}\left(1-\alpha \mu_{1}\right){\left(I-\alpha A\right)}^{-1}j=\beta_{1}z^{\left(1\right)}\propto z^{\left(1\right)}. \end{array}$$

For any damping factor *α*, the main eigenvalues and eigenvectors of a graph suffice to determine the walk centrality vector.

**Theorem 17 .**

The walk centrality vector of a graph is given by the unit vector along

(7) $$\begin{array}{c} y=\overset{p}{\underset{i=1}{\sum }}‍\frac{z^{\left(i\right)}}{1-\alpha \mu_{i}}. \end{array}$$

**Proof**

Spectral decomposition for a matrix **A** with *s* distinct eigenvalues gives **A** = *μ*_1_ **P**_1_ + *μ*_2_ **P**_2_ + ⋯+*μ*_*s*_ **P**_*s*_. If **A** is the adjacency matrix of a graph *G*, then **y** = (**I** − *α* **A**)^−1^ **j** = ∑_*k*=0_^*∞*^ *α* ^*k*^ **A** ^*k*^ **j** = ∑_*i*=1_^*p*^∑_*k*=0_^*∞*^(*αμ*_*i*_)^*k*^ **P**_*i*_ **j** = ∑_*i*=1_^*p*^(1/(1 − *αμ*_*i*_))**P**_*i*_ **j** = ∑_*i*=1_^*p*^(‖**P**_*i*_ **j**‖/(1 − *αμ*_*i*_))**z** ^(*i*)^, since **P**_*i*_ **j** = 0 exactly for the nonmain eigenvalues of **A**.

By Theorem 16, the walk centrality vector depends on *α* and, as *α* tends to 1/*λ*_max⁡_, it approaches the eigenvector centrality. Estrada and Rodríguez-Velázquez suggested a damping factor of 1/*r*! for **A** ^*r*^. The centrality of vertex *i* is then defined as the diagonal entry at position *i* of (**I** + **A** + **A** ^2^/2! + ⋯+**A** ^*r*^/*r*! + ⋯) = *e* ^**A**^ and is referred to as the* Estrada index* [14]. It has also been used as a community detection tool.

### 4.2. Irregularity Scaled-Walk Vertex Representations

In our quest to capture further the realistic possible influences of remote actors, we now consider another vertex ranking parameter, also based on the number of walks of different lengths, which is very sensitive to graph structure. The number of walks *N*_*ℓ*_(*i*) of length *ℓ* in the range 0 to *n* − 1 from a vertex *i* of a *n*-vertex graph can form a row vector *N*(*i*) = (*N*_0_(*i*), *N*_1_(*i*),…, *N*_*n*−1_(*i*)) representing *i*.

**Figure 2:**
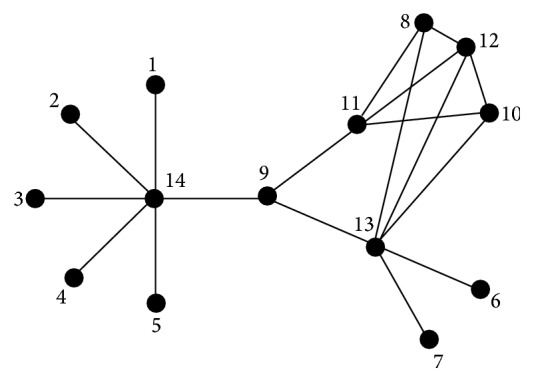
Neighbourhoods of different type for the vertices of highest degree in *G*_14_ on 14 vertices.

For the graph in Figure 2, *N*(11) = (1,4, 13,60,180,774,2452,9928,32882,127860). The same representation may be shared by different vertices as is the case for vertices 8 and 10.

Note that the column vector *N*_*ℓ*_ = (*N*_*ℓ*_(*i*)) = (*N*_*ℓ*_(1), *N*_*ℓ*_(2),…, *N*_*ℓ*_(*n*))^*t*^, giving the number of walks of a particular length *ℓ* from each vertex is **A** ^*ℓ*^ **j**. We note that for a regular graph and a specific length *ℓ*, *N*_*ℓ*_(*i*) is a constant for all vertices *i* and *N*_*ℓ*_ = *N*_*ℓ*_(1)**j**. Therefore vertices of a regular graph are equivalent with respect to the number of walks. We observe also that, for irregular graphs, it is not always the case that the number of walks from the vertices, as *ℓ* increases, ranks the vertices according to the vertex degrees.

The entries of columns **A** **j**, **A** ^2^ **j**,…, **A** ^9^ **j** are the sequences of walks of length 1,2,…, 9.  Table 1 shows the entries from the 8th, 10th, and 11th vertices, respectively, according to the labelling of the graph *G*_14_ in Figure 2. They demonstrate oscillating vertex priorities for small lengths, as shown in Table 1.

However we prove, again using the main eigensystem alone, that there exists a positive integer *R* such that the ranking of the vertices according to the number of walks of length *R* + *k* remains unchanged for all *k* > 0.

**Theorem 18 .**

Let *G* be a connected nonbipartite graph and *N*_*r*_(*i*) the number of walks of length *r* from *i*. Then there exists *R* ∈ *ℤ* ^+^ such that, for all *k* > 0, the ordering of the magnitudes of the number *N*_*R*+*k*_(*i*) of walks of length *R* + *k* from each vertex *i* is independent of *k*.

**Proof**

Let *μ*_1_ = *λ*_1_, *μ*_2_,…, *μ*_*p*_ be the main eigenvalues of *G* with corresponding orthonormal eigenvectors **z** ^(1)^, **z** ^(2)^,…, **z** ^(*p*)^. If **j** = ∑_*i*=1_^*p*^ *β*_*i*_ **z** ^(*i*)^, then

(8) $$\begin{array}{c} A^{q}j=\overset{p}{\underset{i=1}{\sum }}\beta_{i}{\mu_{i}}^{q}z^{\left(i\right)}={\left(\lambda_{\max}\right)}^{q}\overset{p}{\underset{i=1}{\sum }}\beta_{i}{\left(\frac{\mu_{i}}{\lambda_{\max}}\right)}^{q}z^{\left(i\right)}. \end{array}$$

As *q* → *∞*, (*μ*_*i*_/*λ*_max⁡_)^*q*^ → 0 for |*μ*_*i*_| < *λ*_max⁡_, which is the case for all eigenvalues of a nonnegative matrix. Hence for all real *ϵ* > 0, there exists *R* such that

(9) $$\begin{array}{c} \left|\beta_{1}z^{\left(1\right)}-\overset{p}{\underset{i=1}{\sum }}\beta_{i}{\left(\frac{\mu_{i}}{\lambda_{\max}}\right)}^{q}z^{\left(i\right)}\right|<\epsilon j\;\forall q>R. \end{array}$$

Now Perron-Frobenius theorem guarantees that each entry of **z** ^(1)^ is positive. Hence if *ϵ* is chosen to be less than the minimum value of the entries of *β*_1_ **z** ^(1)^, then all the entries of ∑_*i*=1_^*p*^ *β*_*i*_(*μ*_*i*_/*λ*_max⁡_)^*q*^ **z** ^(*i*)^ will be positive for *q* > *R*. Hence there exists *R* such that, for all *q* > *R*, the order of magnitude of the number *N*_*q*_(*i*) of walks of length *q* from each vertex *i* is independent of *q*.

An implicit result in the proof of Theorem 18 is that, as *q* → *∞*, **A** ^*q*^ **j** is proportional to the eigenvector centrality. This suggests a vertex representation which we term the* irregularity scaled-walk centrality*, where a vertex is represented by *SN*(*i*)∶ = (**j**, *α* **A** **j**, *α* ^2^ **A** ^2^ **j**,…, *α* ^*n*−1^ **A** ^*n*−1^ **j**), where *α* = 1/(Δ + 1), Δ being the maximum vertex degree in the network. The vertex priority given by the second entry of *SN*(*i*) is the degree centrality, whereas, by Theorem 18, the entries of *α* ^*ℓ*^ **A** ^*ℓ*^ **j** for larger *ℓ* approach the eigenvector centrality, given in Definition 2.

The overall efforts we considered so far, to make sure that the contribution by distant actors is taken into consideration, involved the concept of distance and walks in graphs. For any network, the walk probability vector and the second entry of the irregularity scaled-walk vertex representation were shown to agree with the degree centrality vector. On the other hand, as *α* → 1/*λ*_max⁡_, the walk centrality was shown to approach the eigenvector centrality. The vectors *α* ^*ℓ*^ **A** ^*ℓ*^ **j** in the irregularity scaled-walk vertex representation also give rankings close to the eigenvector centrality for large *ℓ*. Therefore so far, the centralities that may give different vertex rankings turned out to be covered by the degree, the square, and the walk centralities.

### 4.3. Power Centrality

Now we present a very different concept of* authoritative power*, derived from everyday experience, which goes counter to that governing the spread of data. Dominance of* ego* on others may not depend solely on the number of direct subordinates but also on the extent to which the latter are dependent on* ego* for access to information. The larger the number of connections a subordinate has, the more independent of* ego* it tends to be, reducing* ego*'s power. This contrasts sharply with all the other centrality measures we have considered.

We choose to measure the importance of* ego* (vertex *v*_*i*_) by considering ∑_*j*~*v*_*i*__1/*ρ*_*j*_^2^, for *j* adjacent to *v*_*i*_, which we term* power centrality*, denoted by *Power*(*v*_*i*_) or sometimes by *Power*_*i*_. In this way, the power of* ego*'s neighbours is restricted rather than enhanced by the neighbours' connections.

## 5. Threshold Graphs

In this section we focus on threshold graphs. For this class of graphs, the value of *R* in Theorem 18 has been proved to be 0 in [10]. Therefore for threshold graphs the ranking of the vertices according to the number of walks is independent of the length of the walks.

**Proposition 19 (see [10]).**

For threshold graphs, the ranking of the vertices according to the number of walks of any length is the same as that for the degree centrality.

Proposition 19 asserts that, for threshold graphs, the irregularity scaled-walk vertex representation gives the same vertex priorities as the degree centrality. Degree and eigenvector centralities usually differ as the latter is sensitive to the importance of second neighbours [9]. Moreover, we observe that according to Proposition 19, for threshold graphs, the eigenvector centrality does not add information to the degree centrality.

**Corollary 20 .**

For threshold graphs, the degree and eigenvector centralities rank the vertices in the same way.

Since the contribution to power centrality decreases with increasing degree of a neighbouring vertex, this measure is specifically designed to give a ranking of the vertices possibly different from other centralities. It is surprising that for threshold graphs this is not the case.

**Theorem 21 .**

For threshold graphs, the ranking of the vertices according to the power centrality is the same as that for the degree centrality.

**Proof**

Let *C*(*a*_1_, *a*_2_,…, *a*_*r*_), for *a*_1_ ≥ 2 and |*A*_*i*_| = *a*_*i*_, be a connected threshold graph conformal with the notation used in Figure 1. For *i* ∈ {1,2,…, *r*}, let *v*_*i*_ be a vertex lying in the group *A*_*i*_ having degree *ρ*_*i*_. Recall that *Power*(*v*_*i*_) = ∑_*j*~*v*_*i*__1/*ρ*_*j*_^2^ denotes the power centrality of a vertex *v*_*i*_.

*Case 1*. For odd *r*, we show that *Power*_*r*_ > *Power*_*r*−2_ > ⋯>*Power*_1_ > *Power*_2_ > *Power*_4_ > ⋯>*Power*_*r*−1_ agrees with the degree centrality.

We consider the independent subsets first. For *i* ∈ {2,4,…, *r* − 3}, the neighbourhood *𝒩*(*v*_*i*_) of *v*_*i*_ is *𝒩*(*v*_*i*+2_) ∪ *A*_*i*+1_. Hence

(10)  Powervi=Powervi+2+ai+1ρi+12 thus Power(vi)>Power(vi+2).Now Nv1=A3∪A5∪⋯∪Ar∪A1∖v1,wwwwNv1=Nv2∪A1∖v1therefore Powerv1=Powerv2+a1−1ρ12,wwwwwiwPower(v1)>Power(v2).

For the cliques *A*_*i*_, *i* ∈ {3,5,…, *r*},

(11) N(vi)=N(vi−2)∪Ai−1∪{vi−2}∖{vi}therefore Power(vi)=Power(vi−2)+ai−1ρi−12+1ρi−22−1ρi2,wwwwwiwPower(vi)>Power(vi−2).

This completes the proof for odd *r*.

*Case 2*. For even *r*, we show that *Power*_*r*_ > *Power*_*r*−2_ > ⋯>*Power*_2_ > *Power*_1_ > *Power*_3_ > ⋯>*Power*_*r*−1_ agrees with the degree centrality.

We consider the independent subsets first. For *i* ∈ {1,3,…, *r* − 3}, the neighbourhood *𝒩*(*v*_*i*_) of *v*_*i*_ ∈ *A*_*i*_ is *𝒩*(*v*_*i*+2_) ∪ *A*_*i*+1_. Hence

(12)  Powervi=Powervi+2+ai+1ρi+12, Powervi>Powervi+2,Nv2=Nv1∪A1∖v2therefore Powerv2=Powerv1+a1ρ12−1ρ22,wwwwwiwPower(v2)>Power(v1).

For *i* ∈ {4,6,…, *r*},

(13) Nvi=Nvi−2∪Ai−1∪vi−2∖vitherefore Powervi=Powervi−2+ai−1ρi−12+1ρi−22−1ρi2,wwwwiwwPower(vi)>Power(vi−2).

Thus the result follows.

For the class of threshold graphs, we have shown that the degree centrality alone determines vertex ranking.

**Theorem 22 .**

For a threshold graph, the degree centrality, the eigenvector centrality, the walk probability vector, the walk centrality, each entry of the irregularity scaled-walk centrality, and the power centrality give the same vertex ranking.

## 6. SWIPD-Centrality

Paying attention to detail in structure throughout a network, by considering all distances from* ego*, has revealed that the walk probability vector entries approximate closely the vertex degree centrality. The vertex degree indicates first level priority among the actors of the ability of creating awareness. The walk centrality, for attenuation factor *α* near 1/*λ*_max⁡_, approaches the eigenvector centrality. On the other hand, the vectors *α* ^*ℓ*^ **A** ^*ℓ*^ **j** in the irregularity scaled-walk vertex representation the vectors for *α* = 1/(Δ + 1) approach the degree centrality for small values of *ℓ* and the eigenvector centrality for large values of *ℓ*. The square centrality emphasises the influence of “friends of friends.” In contrast to all these centralities, if the number of second neighbours of* ego* is large, power centrality* usually* reduces* ego*'s importance.

The analysis found in the literature for most of the naturally occurring networks in computer, biological, and social networks places subjective emphasis on some centralities more than on others depending on the aspect that is being studied. We propose a balanced centrality vector, termed **y**_SWIPD_,

(14) ySWIPD∶=γ1A2D−1Aj+γ2D−αDA−1Aj+γ3AD−2j+γ4AD−1Aj,

where the successive terms are the square, the walk, the power, and the degree centralities, respectively. It incorporates the salient features of a network's iterated interactions and can be adjusted to focus on selected aspects of centrality. This centrality is particularly simple to evaluate since it can be expressed solely in terms of an attenuation factor *α*, the adjacency matrix **A**, and the degree diagonal matrix **D**. A balance of intended priorities can be achieved by adjusting the coefficients *γ*_*i*_, for 1 ≤ *i* ≤ 4, of the four terms in SWIPD. The value of the coefficients can give more weight to certain centralities in the ranking of vertices, depending on the intended objectives of the network.

Good performance requires timely delivery of objectives which is achieved by the network structures that determine collaboration (high SWIPD centrality). For regular graphs none of the terms in the SWIPD centrality discriminate among the vertices. The centrality measure SWIPD can be viewed as the extent to which a network is irregular. Only for irregular nonbipartite graphs does the SWIPD centrality give a meaningful ranking of the vertices.

The graph *G* in Figure 2 brings out the salient differences among vertices of the same degree as well as among second neighbours. Recall that to ensure the well definition of the walk centrality vector, we take (*α*)^−1^ > *λ*_max⁡_(**A**). Since in general, the maximum vertex degree Δ of a graph is an upper bound for the maximum eigenvalue *λ*_max⁡_ of **A**, in our examples we choose *α* = 1/(Δ + 1) as in the irregularity scaled-walk vertex representation.

The graph *G*_14_ in Figure 2 has degree sequence {1,1, 1,1, 1,1, 1,3, 3,3, 4,4, 6,6} and the Perron vector for **A** is proportional to {0.277, 0.277, 0.277,  0.277, 0.277, 1.058,  1.058, 2.946, 2.231, 2.946, 3.239,  3.585,  3.825,  1.} The (*γ*_1_ *γ*_2_ *γ*_3_ *γ*_4_)-SWIPD-centrality of the 14-vertex graph *G*_14_ for *γ*_1_ = *γ*_2_ = *γ*_3_ = *γ*_4_ is {0.139674,  0.139674,  0.139674, 0.139674,  0.139674, 0.140477,  0.140477, 0.323735,  0.355234,  0.323735, 0.336275,  0.384093, 0.441474, 0.354914} which gives a ranking that largely agrees with the degree centrality but discriminates among nonsimilar vertices of the same degree. Measures of the (*γ*_1_ *γ*_2_ *γ*_3_ *γ*_4_)-SWIPD-centrality for different weightings *γ*_*i*_ are shown in Table 2.

In *G*_14_, vertex 14 has neighbours totally dependent on it for access to the network. It has higher ranking than vertex 13 for the power centrality even though they have the same degree. It is also interesting to compare the ranking of vertices 14 and 9, which varies considerably with different centralities. As indicated in [9] and as shown above the degree and eigenvector centralities can give contrasting priority as in the case of vertex 14 in the graph *G*_14_. Note that the eigenvector centrality yields an aspect of centrality or status that is not captured by other measures. Vertex 14, for instance, has neighbours with low eigenvector centrality (status) and has low priority according to the eigenvector centrality index. The eigenvector centrality is an appropriate measure when one believes that actors' status is determined by that of their neighbours. This concept of vertex priority is meaningful when social status is highly dependent on that of one's associates [15]. It is noted that the last entry of the irregularity scaled-walk centrality ranks the vertices as the eigenvector centrality as predicted in Theorem 18. From Theorem 22, it follows that the following is true.

**Corollary 23 .**

For threshold graphs, the ranking of the vertices according to the *SWIPD* centrality is independent of the weightings of the contributing terms.

## 7. Conclusion

All the centralities we discussed assign equal importance to all the vertices of a regular graph. On the other extreme of the degree distribution for a prescribed number of vertices, we find antiregular graphs [16] for which only two vertices have the same degree. Antiregular graphs are threshold graphs. A general irregular graph is expected to have different vertex ranking given by the degree, eigenvector, square, and power centralities. For threshold graphs, we discovered the surprising result that the degree centrality, the eigenvector centrality, the power, the walk probability vector, the walk centrality, and each entry of the irregularity scaled-walk centrality give the same ranking. It would be interesting to gauge whether an arbitrary network *G* is sufficiently close to a threshold graph, in which case the degree centrality of the threshold graph would suffice to rank the vertices of *G*.

SWIPD captures intended behaviour and other relation substructural forces that may not be immediately apparent. The measure of arbitrariness in the choice of coefficients is not a weakness. Indeed the ease of determining the values of the matrix expression (14) for SWIPD enables centrality vertex ranking based on diverse network invariants to be compared leading to a clearer picture of the more central vertices and a better understanding of centrality.

## Figures and Tables

**Table 1:** 

Vertex	**j**	**A** **j**	**A** ^2^ **j**	**A** ^3^ **j**	**A** ^4^ **j**	**A** ^5^ **j**	**A** ^6^ **j**	**A** ^7^ **j**	**A** ^8^ **j**	**A** ^9^ **j**
8	1	3	14	44	188	610	2458	8236	31932	109672
10	1	3	14	44	188	610	2458	8236	31932	109672
11	1	4	13	60	180	774	2452	9928	32882	127860

**Table 2:** 

Graph *G*_14_								
Centrality	Rank	Priority						
	1	2	3	4	5	6	7	8
1111SWIPD	13	12	9	14	11	8, 10	6, 7	1, 2, 3, 4, 5
2341SWIPD	13	14	12	9	11	8, 10	6, 7	1, 2, 3, 4, 5
3214SWIPD	13	12	9	11	8, 10	14	6, 7	1, 2, 3, 4, 5
Power	14	13	11	12	8, 10	9	1, 2, 3, 4, 5, 6, 7	
Walk	13	12	14	11	8, 10	9	6, 7	1, 2, 3, 4, 5
Degree	13, 14	11, 12	8, 9, 10	1, 2, 3, 4, 5, 6, 7				
Eigenvector	13	12	11	8, 10	9	6, 7	14	1, 2, 3, 4, 5
Irregularity Scaled-Walk (for high indices)	13	12	11	8, 10	9	6, 7	14	1, 2, 3, 4, 5
